# Multifunctional Hyaluronic Acid-Modified Pterostilbene Nanoliposomes for Enhanced Dermal Delivery and Extracellular Matrix Protection

**DOI:** 10.3390/pharmaceutics18101313

**Published:** 2026-10-16

**Authors:** Zheng Wang, Yumei Fan, Peixue Ling

**Affiliations:** 1 Meyer Bio-Medicine Co., Ltd., Jinan 250022, China; wz252994727@126.com (Z.W.);; 2 Shandong Meimao Pharmaceutical Co., Ltd., Jinan 250022, China; 3 National Glycoengineering Research Center, Shandong University, Jinan 250100, China

**Keywords:** pterostilbene, hyaluronic acid, nanoliposomes, cellular association, skin retention, extracellular matrix

## Abstract

**Background:** Skin aging involves extracellular matrix (ECM) deterioration, including collagen loss and disrupted glycosaminoglycan and proteoglycan homeostasis. Pterostilbene (PTE) has antioxidant and anti-inflammatory activity, but its topical use is limited by poor aqueous solubility and limited skin delivery. We developed hyaluronic acid (HA)-modified, PTE-loaded nanoliposomes (HA-LP-PTE) and evaluated their association with dermal fibroblasts, retention in skin, and effects on ECM-related responses. **Methods:** HA-LP-PTE was prepared by high-pressure microfluidization and assessed for size, morphology, zeta potential, and encapsulation efficiency. Fluorescence microscopy was used to examine fibroblast-associated fluorescence of FITC-labeled HA-LP-PTE and non-HA-modified PTE-loaded liposomes (LP-PTE). Skin retention was assessed using Franz diffusion cells. ECM-related changes were analyzed in UV-irradiated ex vivo skin and fibroblasts using staining techniques and RT-qPCR. **Results:** HA-LP-PTE had a mean diameter of 55.44 ± 0.81 nm, a polydispersity index of 0.120 ± 0.009, and an encapsulation efficiency of 99.34 ± 0.51%. At 4 h, fibroblast-associated fluorescence was higher than with LP-PTE (*p* = 0.040) and was reduced by free HA or incubation at 4 °C. After 12 h, dermal PTE retention was 4.08-fold higher than free PTE and 1.74-fold higher than LP-PTE. HA-LP-PTE increased all six ECM components examined. Chondroitin sulfate and perlecan met the Q criterion for synergy, and several ECM synthesis-related genes were upregulated. **Conclusions:** HA modification improved PTE delivery beyond liposomal encapsulation alone, with greater fibroblast-associated fluorescence and higher PTE retention in viable skin than LP-PTE. ECM and gene-expression findings also indicated matrix-related activity. HA-LP-PTE may enhance topical PTE delivery and attenuate UV-induced ECM loss.

## 1. Introduction

Skin aging is influenced by both intrinsic and extrinsic factors, with changes in the extracellular matrix (ECM) among the main mechanisms of skin aging. [1]. The ECM is a complex three-dimensional network made of collagen, elastin, glycosaminoglycans (GAGs), and proteoglycans (PGs) [2]. It provides physical support and mechanical protection to cells and plays key roles in cell signaling, tissue homeostasis, moisture retention, and elasticity [2,3,4]. During skin aging, the composition, structure, and function of the ECM undergo marked changes. Collagen and elastic fibers degrade, fragment, and lose their normal organization. Also, the balance of GAGs, such as hyaluronic acid (HA) and chondroitin sulfate (CS), is disrupted, along with shortened GAG chains, altered sulfation patterns, and decreased levels of PGs, such as decorin and perlecan [3,4,5,6,7]. Together, these changes cause characteristic signs of skin aging, including wrinkling, sagging, dryness, and loss of elasticity [6,7,8,9]. Therefore, targeting the ECM and promoting the synthesis and appropriate assembly of its key components may be a promising strategy to mitigate skin aging.

Pterostilbene (PTE), a natural stilbene derivative, has attracted attention for its strong antioxidant, anti-inflammatory, and anti-aging activities [10,11]. Previous studies suggest that PTE may reduce skin-aging-related changes through effects on oxidative stress, inflammation, tissue structure, and aging-related signaling pathways [12,13,14]. For example, PTE can effectively help protect skin from damage caused by ultraviolet (UV) rays. In human dermal fibroblasts, PTE has been reported to reduce reactive oxygen species levels, increase lipid synthesis, and promote collagen production, supporting its potential as an anti-photoaging agent [13]. It can also suppress the release of proinflammatory cytokines, thereby reducing structural skin damage associated with chronic inflammation [15]. However, the topical use of PTE is limited by its physicochemical properties, including low aqueous solubility, low bioavailability, and susceptibility to light-induced trans-to-cis isomerization, all of which can reduce its bioactivity [16,17]. To address these limitations, researchers have developed several nanoscale delivery systems, including nanoemulsions, nanomicelles, and nanoparticles, to improve the solubility, stability, and bioavailability of PTE [18,19,20,21,22]. Despite these efforts, current skincare delivery systems may still face skin-safety concerns, potential irritation from residual organic solvents, limited industrial feasibility, and physicochemical instability [20,21,22]. Therefore, developing a PTE delivery system that combines high safety, simple preparation, strong stability, and effective promotion of ECM synthesis is critically important for future application potential.

HA is a linear polysaccharide naturally present in the skin and a major ECM component in the dermis [5]. It has been reported to mitigate UV-induced skin aging [23,24]. As a key ECM component, HA is highly hydrophilic, forms hydrated viscoelastic networks, and is biocompatible and biodegradable, making it an attractive controlled-release material for biomedicine and cosmetics [25]. Studies have shown that integrating HA with drug-loaded liposomes can prolong drug release and greatly improve biosafety and stability [26,27,28]. HA may also help liposomes interact with CD44-expressing skin cells, like dermal fibroblasts, improving cellular association and localization of the loaded bioactive compounds within the dermis [29,30]. However, it remains unclear whether HA surface modification directly improves the interaction of PTE-loaded liposomes with dermal fibroblasts under these conditions. We therefore hypothesized that HA modification would combine the penetration and encapsulation benefits of liposomes with the hydration- and ECM-related biological functions of HA. HA modification might also improve PTE delivery and dermal retention through HA-associated cellular interactions that may involve CD44. Compared with conventional PTE-loaded liposomes, HA-modified liposomes may have better dispersion stability, greater dermal retention, and stronger ECM-restoring effects in UV-damaged skin.

In this study, HA-modified PTE-loaded nanoliposomes (HA-LP-PTE) were developed as a topical delivery system to enhance dermal PTE retention and to evaluate their effects on UV-induced ECM alterations in an ex vivo skin model. The physicochemical properties, encapsulation performance, stability, and skin-delivery behavior of the nano-system were evaluated. To assess the role of HA modification in cellular interactions, cell-associated fluorescence of FITC-labeled LP-PTE and HA-LP-PTE in primary human dermal fibroblasts was compared over time, after competition with free HA, and at low temperature. To clarify the effects of the formulation components, LP-PTE and HA-LP-PTE were compared in the cellular-association and skin-retention experiments, while free PTE and HA-LP free were used as controls in the skin-retention and ECM experiments. The effects of HA-LP-PTE on the ECM were further examined in UV-irradiated ex vivo skin by measuring collagen I, elastin, HA, CS, decorin, and perlecan. The study aimed to investigate whether HA modification could improve local PTE delivery and attenuate ECM alterations following short-term UV irradiation.

## 2. Materials and Methods

### 2.1. Chemicals and Solvents

PTE (99.8% purity) was obtained from Beri Pharma Co., Ltd. (Zhuhai, China), and phosphatidylcholine (PtdCho) from Nippon Surfactant Industries Co., Ltd. (Tokyo, Japan). Caprylic/capric triglyceride (CCT) was obtained from QemibondChem PTE. Ltd. (Singapore), sodium hyaluronate from Guantianxia (Jinan, China) Biotechnology Co., Ltd. (Linyi, China), and 1,2-hexanediol from Ashland LLC (Wilmington, DE, USA). Phosphate-buffered saline (PBS) was purchased from Solarbio Science & Technology Co., Ltd. (Beijing, China). Fluorescein isothiocyanate (FITC), vitamin C, and vitamin E, as well as HPLC-grade acetonitrile and methanol, were procured from Sigma-Aldrich (St. Louis, MO, USA). Paraformaldehyde was obtained from Biosharp Life Sciences (Hefei, China). The primary antibody against collagen I was obtained from Cell Signaling Technology (Danvers, MA, USA). Biotin-conjugated hyaluronan-binding protein was acquired from Amsbio (Abingdon, UK). Primary antibodies against perlecan, decorin, chondroitin sulfate, and elastin were procured from Abcam (Cambridge, UK). The ex vivo skin model and its corresponding culture medium were both obtained from Shaanxi Biocyto General Testing Technology Co., Ltd. (Xi’an, China). Primary human dermal fibroblasts and their culture media were obtained from Lifeline Cell Technology (Frederick, MD, USA). A RNeasy Mini Kit (Qiagen, Hilden, Germany) was used for total RNA extraction. The PrimeScript RT Reagent Kit and TB Green Premix Ex Taq (Takara, Kyoto, Japan) were used for reverse transcription and quantitative PCR, respectively. Ultrapure water was prepared using a Milli-Q water purification system (Millipore Sigma, Burlington, MA, USA) and had a resistivity of 18.2 MΩ·cm at 25 °C.

### 2.2. Preparation of LPs

The HA-LP-PTE formulation was developed based on preliminary formulation screening conducted during the development of the patented liposomal technology [31]. The key variables investigated included phospholipid content, HA concentration, and oil-phase composition, with prototype formulations evaluated in terms of particle size, polydispersity index (PDI), and PTE skin retention. HA-LP-PTE was subsequently prepared by high-pressure microfluidization following the patented procedure, with minor modifications. Briefly, a homogeneous lipid matrix was prepared by heating PtdCho and CCT (at a mass ratio of 7:20) under magnetic stirring at 75 °C for 30 min. After cooling to 55 °C, PTE was added to the lipid melt. The mixture was stirred at 400 rpm under nitrogen until PTE was fully dissolved and formed the lipid phase. Under continuous stirring, the lipid phase was cooled to 25 °C and then injected at 10–60 mL/min into an aqueous phase containing HA and 1,2-hexanediol at the same temperature. The primary emulsion was stirred at 200 rpm for 20 min to complete hydration and was then processed for three cycles at 20,000 psi using a microfluidizer (M-110P, Microfluidics International Corporation, Westwood, MA, USA). A circulating water bath kept the process temperature at 25 ± 1 °C. The final product was a semitransparent, light-yellow nanodispersion.

As controls, HA-modified blank liposomes without PTE (HA-LP free) were prepared using the same procedure but without PTE. PTE-loaded liposomes without HA modification (LP-PTE) were prepared using the same procedure, except that HA was left out of the aqueous phase. The nanodispersions were then stored at 4 °C in the dark until characterization. Table 1 lists the composition of each formulation.

### 2.3. Physicochemical Characterization of LPs

The prepared liposomes were characterized for mean particle size, PDI, zeta potential, morphology, and HA–lipid interfacial interactions. The mean particle size (Z-average) and PDI were measured by dynamic light scattering with 1% (*w*/*w*) aqueous dispersions. Zeta potential was determined by electrophoretic light scattering with 10% (*w*/*w*) aqueous dispersions. The dispersant refractive index was set to 1.33, and the equilibrium time was 60 s. All measurements were performed at 25 °C with a BeNano 180 Zeta Pro particle analyzer (Bettersize Instruments Ltd., Dandong, China).

Cryo-transmission electron microscopy (cryo-TEM) was used to examine the morphology of HA-LP free using a Themis 300 microscope (Thermo Fisher Scientific, Waltham, MA, USA) operated at 120 kV. A drop of the HA-LP-PTE dispersion was placed on a copper grid coated with a lacey carbon film, and excess liquid was removed with filter paper. The grid was quickly frozen in liquid ethane and moved to the cryo-holder under liquid nitrogen. Images were acquired at magnifications of 20,000 to 120,000×.

Fourier transform infrared spectroscopy (FTIR) was used to analyze possible interactions between HA and PtdCho in HA-LP free. Prior to FTIR analysis, the samples were pre-frozen at −28 °C for 12 h and subsequently lyophilized at −20 °C for 24 h using a freeze dryer (BILON-HFD-01, Shanghai Bilon Instrument Manufacturing Co., Ltd., Shanghai, China). The temperature was then gradually increased to room temperature at a rate of 5 °C/h. Freeze-dried HA, PtdCho, and HA-LP free samples were mixed with dry KBr, pressed into pellets, and analyzed using a Nicolet iS50 FTIR spectrometer (Thermo Fisher Scientific, Madison, WI, USA) over the range of 4000–500 cm^−1^ at a spectral resolution of 4 cm^−1^. The spectra were corrected for baseline and atmospheric effects before characteristic band shifts were compared.

The preliminary physicochemical stability of HA-LP-PTE and LP-PTE was evaluated under different storage conditions. After preparation, the formulations were equilibrated at room temperature for 24 h, and the resulting measurements were used as the baseline. The samples were subsequently stored in sealed plastic vials at 4 °C, room temperature, −20 °C, or 45 °C without additional light protection for up to 30 days. The 45 °C condition was included as a preliminary accelerated stability challenge. Visual appearance, pH, and mean particle size were assessed after 7 and 30 days of storage. The pH and particle size were determined using 10% and 1% (*w*/*w*) aqueous dispersions, respectively.

### 2.4. Analysis of PTE Encapsulation Efficiency and Drug Loading

PTE concentrations in all samples were measured using a validated high-performance liquid chromatography (HPLC) method. The encapsulation efficiency (EE) and drug loading (DL) of the LPs were measured by ultrafiltration–centrifugation followed by HPLC analysis.

#### 2.4.1. HPLC Analysis

PTE was quantified using an HPLC method adapted from a previous study [32] with minor modifications. An Agilent ZORBAX SB-C18 column (4.6 × 250 mm, 5 μm) was used. Isocratic elution was performed using 0.1% phosphoric acid in water (mobile phase A) and acetonitrile (mobile phase B) at a volume ratio of 40:60 (A:B, *v*/*v*). The column temperature was maintained at 30 °C, with an injection volume of 5 μL and detection at 306 nm. Each run lasted 10 min. A standard curve was made by plotting peak area against PTE concentration under these conditions.

#### 2.4.2. Analysis of Encapsulation Efficiency and Drug Loading

For each formulation, 500 mg was dispersed in ultrapure water to a final volume of 10 mL, giving a 5% (*w*/*v*) dispersion. An aliquot (0.2 mL) was diluted to 10 mL with 90% acetonitrile and vortexed for 5 min. The solution was then passed through a 0.22 μm PTFE membrane. PTE in the filtrate was measured by HPLC and used to determine the total drug content (W_total). A 0.5 mL aliquot of each 5% (*w*/*v*) LP-PTE or HA-LP-PTE dispersion was placed in a 3 kDa molecular-weight-cutoff ultrafiltration tube and centrifuged using an H1750 centrifuge (Hunan Xiangyi Laboratory Instrument Development Co., Ltd., Changsha, China) equipped with a No. 1 rotor at 7000 rpm for 20 min at room temperature. Then, 0.2 mL of the filtrate was diluted to 10 mL with 90% acetonitrile, vortexed for 5 min, and filtered through a 0.22 μm PTFE membrane. The PTE concentration in this filtrate represented the free drug content (W_free) and was measured by HPLC. Representative HPLC chromatograms of total PTE (W_total) and free PTE (W_free) in HA-LP-PTE, used for the calculation of EE and DL, are provided in Supplementary Figure S1. EE and DL were calculated with the following equations:

(1) EE(%) = (W_total − W_free)/W_total × 100%,

(2) DL(%) = (W_total − W_free)/W_LP × 100%,

where W_LP represents the total mass of the liposomal formulation used. The PTE content (W) was calculated as follows:

(3) W = (S_sample × V_sample × m_reference)/(S_reference × V_reference × m_sample) × F × 100%

where S is the peak area, V is the dilution volume, m is the mass, and F is the purity factor of the reference standard (99.8%).

### 2.5. Evaluation of Cellular Association of FITC-Labeled LPs

Cellular association of FITC-labeled LPs was evaluated in primary human dermal fibroblasts [33]. FITC-labeled HA-LP-PTE and LP-PTE were prepared by adding FITC directly during liposome preparation. After preparation, the FITC-labeled liposomal dispersions were passed through a 0.22 μm membrane filter to remove precipitated FITC particles. The filtrates containing the FITC-labeled liposomes were collected for subsequent cellular association experiments. A preliminary MTT assay was used to identify a noncytotoxic working concentration, and 0.1% FITC-labeled formulations were selected. Fibroblasts were seeded in 96-well plates at 1 × 10^4^ cells/well and cultured overnight until they reached about 60–70% confluence. Cells were then treated with 0.1% FITC-labeled HA-LP-PTE or LP-PTE at 37 °C. To test HA-associated uptake, the cells were preincubated with free HA at 5 mg/mL for 1 h before treatment with FITC-labeled HA-LP-PTE. To assess energy-dependent uptake, cells treated with FITC-labeled HA-LP-PTE were incubated at 4 °C. At 0, 2, 4, and 6 h, the cells were washed three times with cold PBS, fixed with 4% paraformaldehyde, and stained with DAPI. Fluorescence images were taken with a fluorescence microscope (MIX60-FL, Guangzhou Ming-Mei Technology Co., Ltd., Guangzhou, China). DAPI and FITC fluorescence was recorded in the blue and green channels, respectively. Cellular fluorescence intensity was quantified in ImageJ (version 1.53t). Background fluorescence was subtracted from the measured MFI to give the net MFI. To correct for differences at baseline, the 0 h net MFI was subtracted from each later time point. The resulting baseline-adjusted MFI values were used for comparisons among groups.

### 2.6. Evaluation of In Vitro Skin Retention

The skin-retention experiment used vertical Franz diffusion cells (RT800, Ruituo Instrument Equipment Co., Ltd., Shenzhen, China). Frozen Bama miniature pig skin (thickness 0.55–0.75 mm) was obtained from the Beijing Fuhao Experimental Animal Breeding Center (Beijing, China; Permit No. 00038561). After thawing and rinsing with saline, skin integrity was assessed using a VapoMeter (SWL5201, Delfin, Finland), and only samples with transepidermal water loss (TEWL) values below 15 g/m^2^/h were used. The skin was carefully placed between the donor and receptor chambers with the stratum corneum facing the donor side. The effective diffusion area was 1.13 cm^2^. The receptor chamber contained 8 mL of phosphate-buffered saline, was kept at 37 ± 1 °C with a water jacket, and was stirred at 600 rpm. Air bubbles were fully removed to ensure direct contact between the dermis and the receptor medium.

Then, 0.1 g of each test formulation—0.019% PTE, 3.8% HA-LP-PTE, or 3.8% LP-PTE—was evenly applied to the skin surface in the donor compartment. The skin-retention experiment was performed in triplicate (*n* = 3). After 12 h, the remaining formulation was gently removed from the skin. TEWL was measured again to confirm that skin integrity was maintained. The SC was then removed by tape stripping. The remaining viable epidermis and dermis were fully minced, and the retained PTE was extracted with 80% methanol. The PTE content of the skin extracts was quantified using the HPLC method described in Section 2.4.1.

### 2.7. Evaluation of ECM Components in Ex Vivo Skin

The ex vivo skin samples, derived from the abdominal skin of a single 28-year-old female donor, were divided into six groups: blank control (BC), negative control (NC), positive control (PC; 100 μg/mL vitamin C and 7 μg/mL vitamin E), PTE (0.004%, *w*/*w*), HA-LP free (0.8%, *w*/*w*), and HA-LP-PTE (0.8%, *w*/*w*). Six separate skin explants were used for each group. After an initial 2-day culture period, all groups except the BC group were exposed daily to UVA (30 J/cm^2^) and UVB (50 mJ/cm^2^) irradiation for 4 days. After each irradiation session, the NC, PTE, HA-LP free, and HA-LP-PTE groups received fresh culture medium, while the PC group received medium with vitamins C and E. The PTE, HA-LP free, and HA-LP-PTE groups also received topical applications of their respective formulations. All groups, except BC and NC, received daily topical treatment during the three days without irradiation. Finally, the skin samples were fixed in 4% paraformaldehyde, embedded, and sectioned for analysis of ECM components by immunofluorescence, immunohistochemistry, and biotin-based staining.

#### 2.7.1. Immunofluorescence Staining

Sections were deparaffinized in xylene, rehydrated through a graded ethanol series, and subjected to high-pressure heat-mediated antigen retrieval in sodium citrate buffer. After endogenous peroxidase was blocked with 3% H_2_O_2_ and nonspecific binding sites were blocked with serum, the sections were incubated overnight at 4 °C with primary antibodies against collagen I, chondroitin sulfate, perlecan, and decorin. After washing, the sections were incubated with fluorescent secondary antibodies for 1 h at room temperature. Nuclei were stained with Hoechst dye, and the slides were mounted with antifade medium. Fluorescence images were acquired within 24 h using a BX43 microscope (Olympus, Tokyo, Japan).

#### 2.7.2. Immunohistochemical Staining

The paraffin-embedded sections were incubated at 70 °C for 4 h, deparaffinized in xylene, and rehydrated through a graded ethanol series. Antigen retrieval used high-pressure heating in 0.01 M sodium citrate buffer. After endogenous peroxidase activity and nonspecific binding were blocked with 3% H_2_O_2_ and serum, respectively, the sections were incubated overnight at 4 °C with a primary antibody against elastin. After washing with PBS, the sections were incubated with a secondary antibody for 1 h and with ABC reagent for 30 min at room temperature. Color was developed with DAB, and the sections were counterstained with hematoxylin. The slides were then dehydrated, cleared, and mounted. The stained sections were visualized and photographed with an upright microscope (BX53, Olympus Corporation, Tokyo, Japan).

#### 2.7.3. Biotin-Based Staining

The staining procedure was adapted from Bodendorf et al. [34], with modifications. Briefly, the paraffin sections were heated at 70 °C for 4 h, deparaffinized in xylene, and rehydrated through a graded ethanol series. After washing with PBS, the sections were treated with hyaluronidase at 37 °C in a humidified chamber (40–60% relative humidity) for 30 min. The sections were then incubated at room temperature with 100 µL of hyaluronan-binding protein for 1 h. After further PBS washes, endogenous peroxidase activity was blocked with 3% H_2_O_2_, and nonspecific binding sites were blocked with serum. The sections were incubated overnight at 4 °C with the primary antibody and then for 1 h at room temperature with the secondary antibody. Nuclei were counterstained with Hoechst dye, and the slides were mounted with antifade medium. The sections were viewed by fluorescence microscopy (BX43, Olympus Corporation, Tokyo, Japan) within 24 h.

### 2.8. Effects of HA-LP-PTE on ECM Synthesis-Related Gene Expression

Primary human dermal fibroblasts were cultured at 37 °C in a humidified atmosphere containing 5% CO_2_. At 80% confluence, the cells were trypsinized and seeded in 12-well plates for 48 h to allow attachment. The cells were then treated with 0.01% HA-LP-PTE for 24 h. After treatment, the supernatant was removed, and the cells were washed twice with ice-cold PBS. Total RNA was extracted from the cell lysates using a RNeasy Mini Kit, and the RNA was reverse-transcribed into cDNA. The expression levels of ECM synthesis-related genes were measured by real-time quantitative PCR (qPCR) using a QuantStudio 5 Real-Time PCR System (Applied Biosystems, Foster City, CA, USA) and SYBR Green chemistry. Relative gene expression was calculated with the 2^–ΔΔCt^ method. Table 2 lists the primer sequences.

### 2.9. Statistical Analysis

Data are shown as the mean ± standard deviation (SD). Statistical analyses were performed using R (version 4.4.2), and graphs were generated using GraphPad Prism (version 9.5). Normality and homogeneity of variance were assessed before test selection. For two-group comparisons, Student’s t-test was used when normality and equal variance were satisfied, Welch’s t-test when normality but unequal variance were satisfied, and the Wilcoxon rank-sum test when normality was not satisfied. For multiple-group comparisons, one-way ANOVA was used when normality and equal variance were satisfied, Welch’s one-way ANOVA when normality but unequal variance were satisfied, and the Kruskal–Wallis test when normality was not satisfied. When the overall test was significant, post hoc comparisons were performed using Dunnett’s test for comparisons between treatment groups and the control group, Tukey’s HSD test or the Games–Howell test for pairwise comparisons among groups depending on variance homogeneity, and Dunn’s test for nonparametric data. The specific statistical method used for each experiment is described in the corresponding figure or table legend. A value of *p* < 0.05 was considered statistically significant. All experiments included at least three independent replicates.

## 3. Results

### 3.1. Physicochemical Characterization of the Nanocarriers

Dynamic light scattering showed that all three liposomal formulations had particle sizes of about 52–56 nm and PDI values below 0.3 (Table 3), indicating that the preparation method produced relatively uniform nanoscale dispersions [41]. HA-LP free, LP-PTE, and HA-LP-PTE had mean particle sizes of 54.15 ± 1.03, 52.80 ± 0.29, and 55.44 ± 0.81 nm, respectively. The corresponding PDI values were 0.157 ± 0.020, 0.089 ± 0.017, and 0.120 ± 0.009, indicating that PTE loading and HA incorporation did not produce a broad particle-size distribution under the preparation conditions used.

The three formulations differed in zeta potential. HA-LP free and LP-PTE showed values of −2.49 ± 0.13 and −4.73 ± 0.15 mV, respectively, whereas HA-LP-PTE reached −15.12 ± 0.22 mV. Thus, incorporation of both HA and PTE was associated with a more negative surface charge. The PDI nevertheless remained low, with no obvious loss of uniformity.

**Figure 1:**
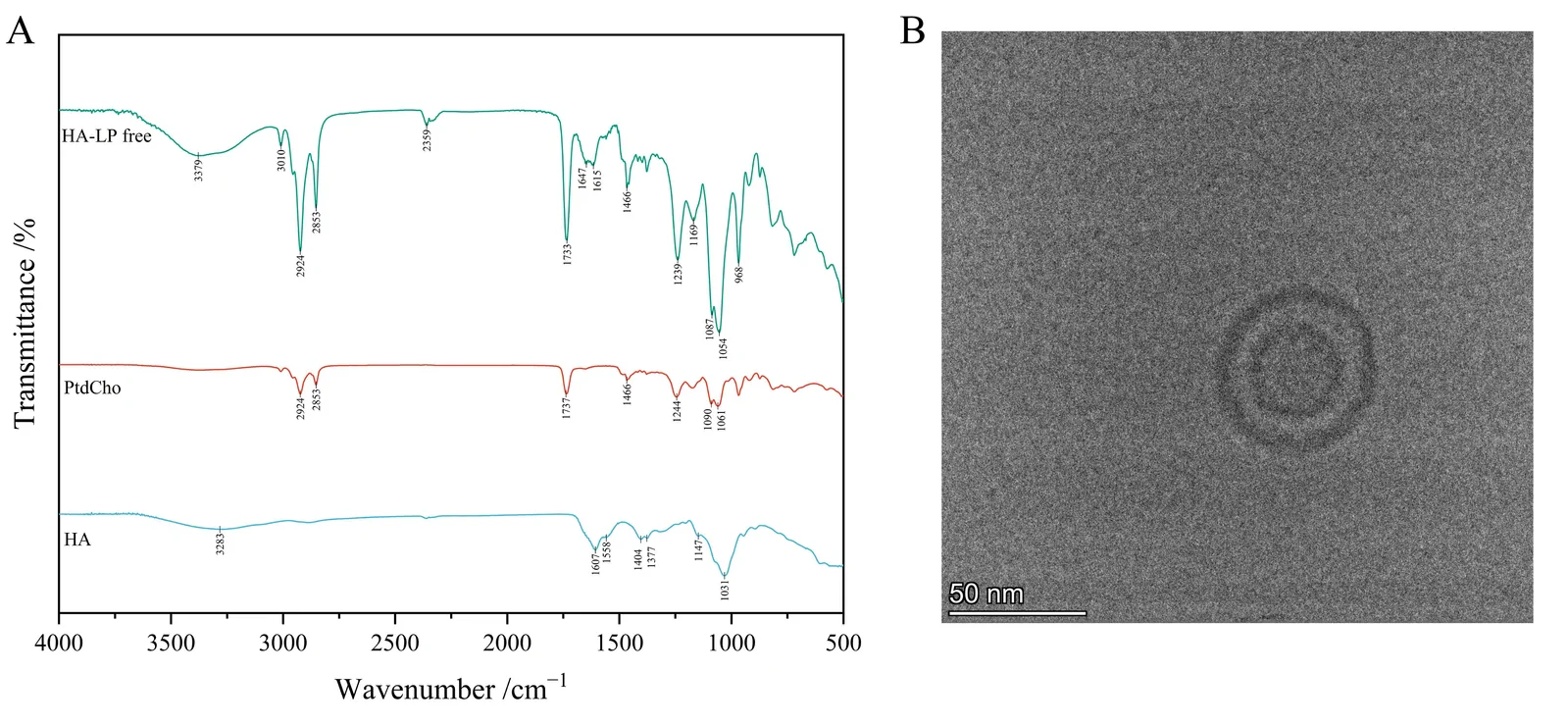
FTIR and Cryo-TEM characterization of HA-modified liposomes. (**A**) FTIR spectra of HA, PtdCho, and HA-LP free. (**B**) Cryo-TEM image of hyaluronic acid-modified PTE-loaded liposomes (HA-LP-PTE). Scale bar: 50 nm.

The FTIR spectra of HA, PtdCho, and HA-LP free were compared to investigate potential HA–lipid interactions (Figure 1A). Native HA exhibited a broad band near 3283 cm^−1^, attributable to hydroxyl and/or amino stretching. The bands at 1607 and 1558 cm^−1^ were assigned to carboxylate and amide vibrations, respectively. Bands at 1147 and 1031 cm^−1^ were associated with C–O–C and C–O stretching. In the HA-LP free, these HA-related bands shifted to about 3379, 1647, 1615, 1169, and 1054 cm^−1^, suggesting possible changes in the local chemical environment of HA in the liposomal formulation. The main PtdCho methylene stretching bands at 2924 and 2853 cm^−1^ showed only minor changes in HA-LP free. In contrast, shifts were observed in bands associated with the polar phospholipid groups. The ester carbonyl band shifted from 1737 to 1733 cm^−1^, the phosphate-related band shifted from 1244 to 1239 cm^−1^, and the P–O–C/C–O–P or phosphocholine-related bands shifted from 1090 and 1061 cm^−1^ to 1087 and 1054 cm^−1^, respectively. This pattern of relatively unchanged acyl-chain bands and shifted polar-headgroup bands suggests that HA may interact with the hydrophilic interfacial region of the PtdCho liposomes.

Cryo-TEM images showed that HA-LP-PTE particles were approximately spherical with smooth contours (Figure 1B), consistent with the nanoscale particle sizes determined by DLS. Together, the DLS, zeta-potential, FTIR, and cryo-TEM results characterize the physicochemical properties of the liposomal systems and are consistent with HA-associated interfacial changes.

The preliminary physicochemical stability of HA-LP-PTE and LP-PTE was also evaluated under different storage conditions for 30 days (Supplementary Table S1). At 4 °C, HA-LP-PTE maintained its light-yellow appearance without visible phase separation, and its mean particle size increased moderately from 55.44 to 60.58 nm. At room temperature, the particle size increased to 70.09 nm after 30 days. Under elevated-temperature stress (45 °C), HA-LP-PTE exhibited substantial particle size growth, reaching 279.80 nm after 30 days, but showed no visible phase separation. In contrast, LP-PTE underwent visible phase separation under the same conditions. Both formulations also exhibited pronounced particle size increases after storage at −20 °C. Overall, HA-LP-PTE showed relatively limited particle size changes at 4 °C, suggesting that refrigerated storage may be preferable among the investigated conditions.

### 3.2. Drug Loading and Encapsulation Efficiency of PTE-Loaded Liposomes

DL and EE are important parameters for evaluating nano-delivery systems. Both PTE-loaded formulations had high EE values, with 97.67 ± 0.75% for LP-PTE and 99.34 ± 0.51% for HA-LP-PTE. The corresponding DL values were 0.486 ± 0.013% and 0.480 ± 0.018%, respectively (Table 3). The DL values of the two formulations were numerically similar and close to the preset PTE feed concentration of 0.5%. The initial PTE feed concentration was set at 0.5% based on a clinical study in which a topical cream containing 0.4% PTE had significant anti-aging and skin-brightening effects in healthy volunteers [42]. Therefore, the DL value in this study reflects the PTE loading achieved at the predefined feed concentration rather than the maximum loading capacities of the liposomal systems. Together with the high EE values, these results suggest satisfactory PTE incorporation and retention during formulation preparation.

### 3.3. Cellular Association of FITC-Labeled LPs in Fibroblasts

**Figure 2:**
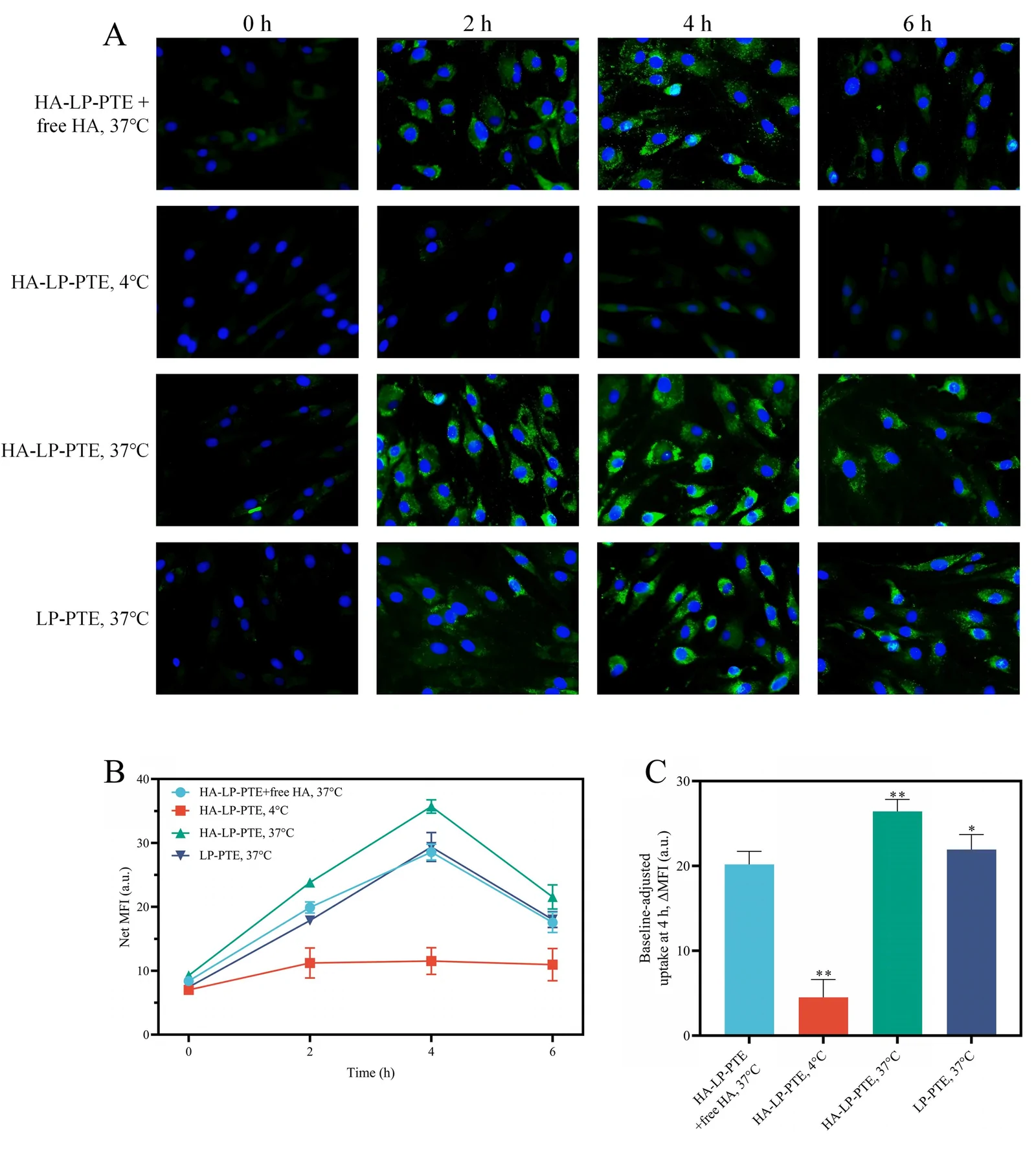
Cellular association of FITC-labeled liposomes within primary human dermal fibroblasts. (**A**) Representative fluorescence images showing cell-associated FITC fluorescence over time under four conditions: pterostilbene (PTE)-loaded liposomes (LP-PTE) at 37 °C; hyaluronic acid-modified PTE-loaded liposomes (HA-LP-PTE) at 37 °C; HA-LP-PTE after pretreatment with free HA (5 mg/mL, 1 h); and HA-LP-PTE at 4 °C. Images were acquired at 0, 2, 4, and 6 h. Nuclei were stained with DAPI (blue), and FITC-labeled liposomes are shown in green. (**B**) Quantitative analysis of net mean fluorescence intensity (net MFI) over time under the four treatment conditions at 0, 2, 4, and 6 h. (**C**) Baseline-adjusted MFI at 4 h under the same four conditions. One-way ANOVA followed by Tukey’s HSD post hoc test was used for multiple comparisons among groups at 4 h. Statistical significance: * *p* < 0.05 and ** *p* < 0.01 versus HA-LP-PTE at 37 °C.

A preliminary MTT assay showed no reduction in fibroblast viability after treatment with 0.1% FITC-labeled formulations. Viability was 101.32 ± 2.23% for HA-LP-PTE and 102.38 ± 3.10% for LP-PTE (Supplementary Table S2). This concentration was therefore used in the fluorescence experiments. Representative images and quantitative results are shown in Figure 2. Fluorescence microscopy showed that cell-associated fluorescence increased from 0 to 4 h and then decreased at 6 h. The highest fluorescence intensity was observed at 4 h (Figure 2A,B). At 37 °C, the raw net MFI at 4 h was higher for HA-LP-PTE than for LP-PTE (35.713 ± 1.047 vs. 29.375 ± 2.260). Because the treatment groups had different initial fluorescence levels, the primary comparison at 4 h used baseline-adjusted MFI, as described in Section 2.5 (Figure 2C). HA-LP-PTE at 37 °C had a baseline-adjusted MFI of 26.444, which was modestly but significantly higher than that of LP-PTE at 37 °C (*p* = 0.040). Excess free HA reduced the baseline-adjusted MFI of HA-LP-PTE to 20.185 (*p* = 0.0093), and incubation at 4 °C reduced it to 4.513 (*p* < 0.001) relative to HA-LP-PTE at 37 °C.

### 3.4. In Vitro Skin Retention of PTE from Different Formulations

**Figure 3:**
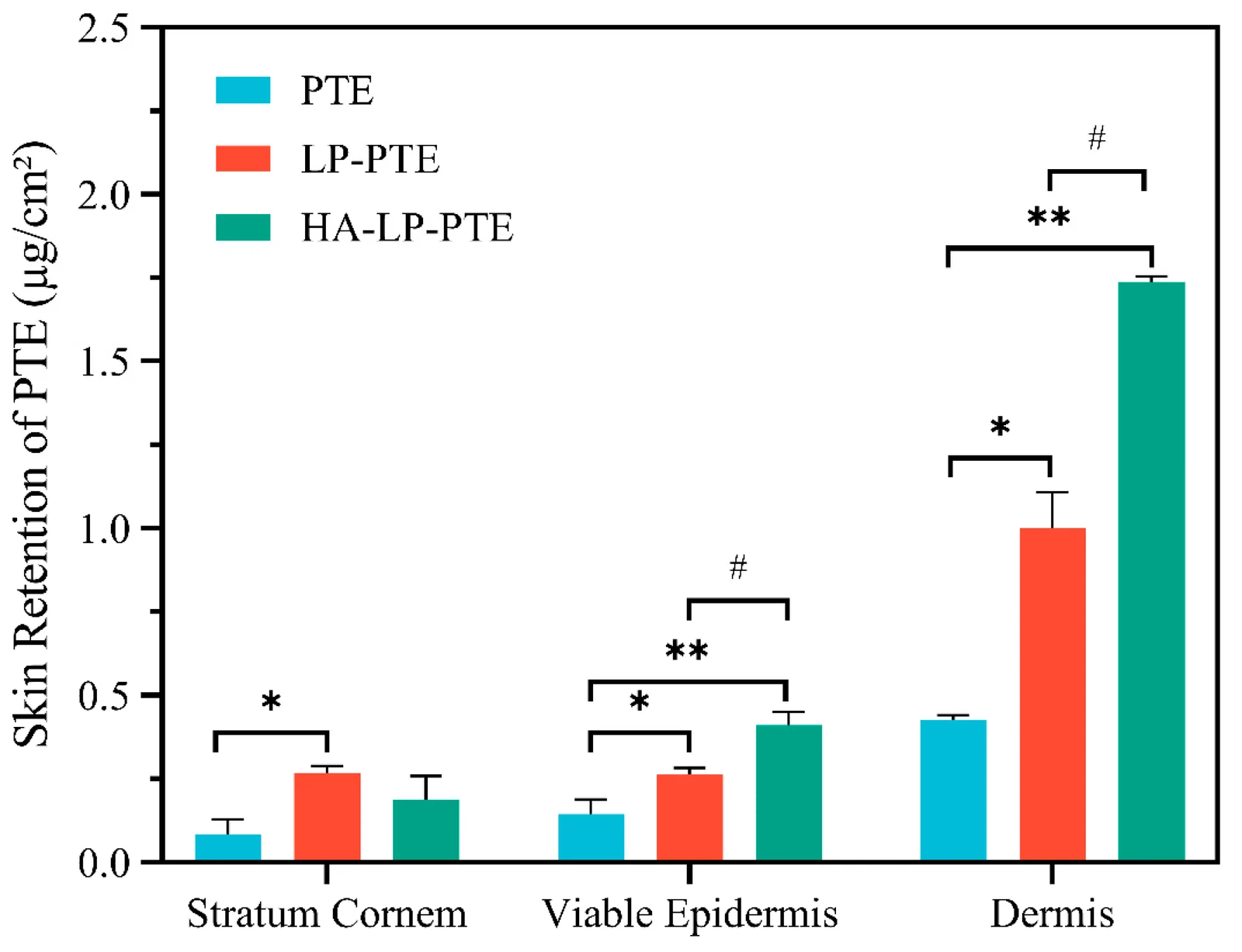
In vitro skin retention of pterostilbene (PTE) in different skin layers after 12 h of permeation from three formulations: free PTE, PTE-loaded liposomes without hyaluronic acid modification (LP-PTE), and hyaluronic acid-modified PTE-loaded liposomes (HA-LP-PTE). Welch’s one-way ANOVA followed by the Games–Howell post hoc test was used for comparisons among formulations within each skin layer. Statistical significance: * *p* < 0.05 and ** *p* < 0.01 versus free PTE; # *p* < 0.05 versus LP-PTE.

Skin retention of PTE from free PTE, LP-PTE, or HA-LP-PTE was evaluated using ex vivo skin. Figure 3 shows that all formulations produced measurable PTE retention in the stratum corneum (SC), viable epidermis, and dermis after 12 h of application, but the amounts and distributions differed markedly among the formulations.

Free PTE showed low retention in all skin layers: 0.084 ± 0.055 μg/cm^2^ in the SC, 0.144 ± 0.054 μg/cm^2^ in the viable epidermis, and 0.426 ± 0.018 μg/cm^2^ in the dermis. Compared with free PTE, LP-PTE significantly increased PTE deposition in the SC, viable epidermis, and dermis to 0.265 ± 0.029, 0.262 ± 0.024, and 1.000 ± 0.130 μg/cm^2^, respectively (all *p* < 0.05). These data show that liposomal encapsulation was associated with greater skin deposition of PTE.

HA-LP-PTE further increased PTE accumulation in the viable skin layers compared with LP-PTE. In the viable epidermis, HA-LP-PTE produced PTE retention of 0.410 ± 0.050 μg/cm^2^, about 2.85-fold higher than free PTE (*p* < 0.01) and 1.56-fold higher than LP-PTE (*p* < 0.05). The largest increase occurred in the dermis, where HA-LP-PTE delivered 1.738 ± 0.018 μg/cm^2^ PTE. This value was 4.08-fold higher than that of free PTE (*p* < 0.01) and 1.74-fold higher than that of LP-PTE (*p* < 0.05). These results suggest that HA modification kept the penetration benefit of the liposomal system and also increased PTE deposition in deeper skin, especially in the dermis.

### 3.5. Impact of Loaded and Blank Nanocarriers on ECM Components in UV-Irradiated Ex Vivo Skin

**Figure 4:**
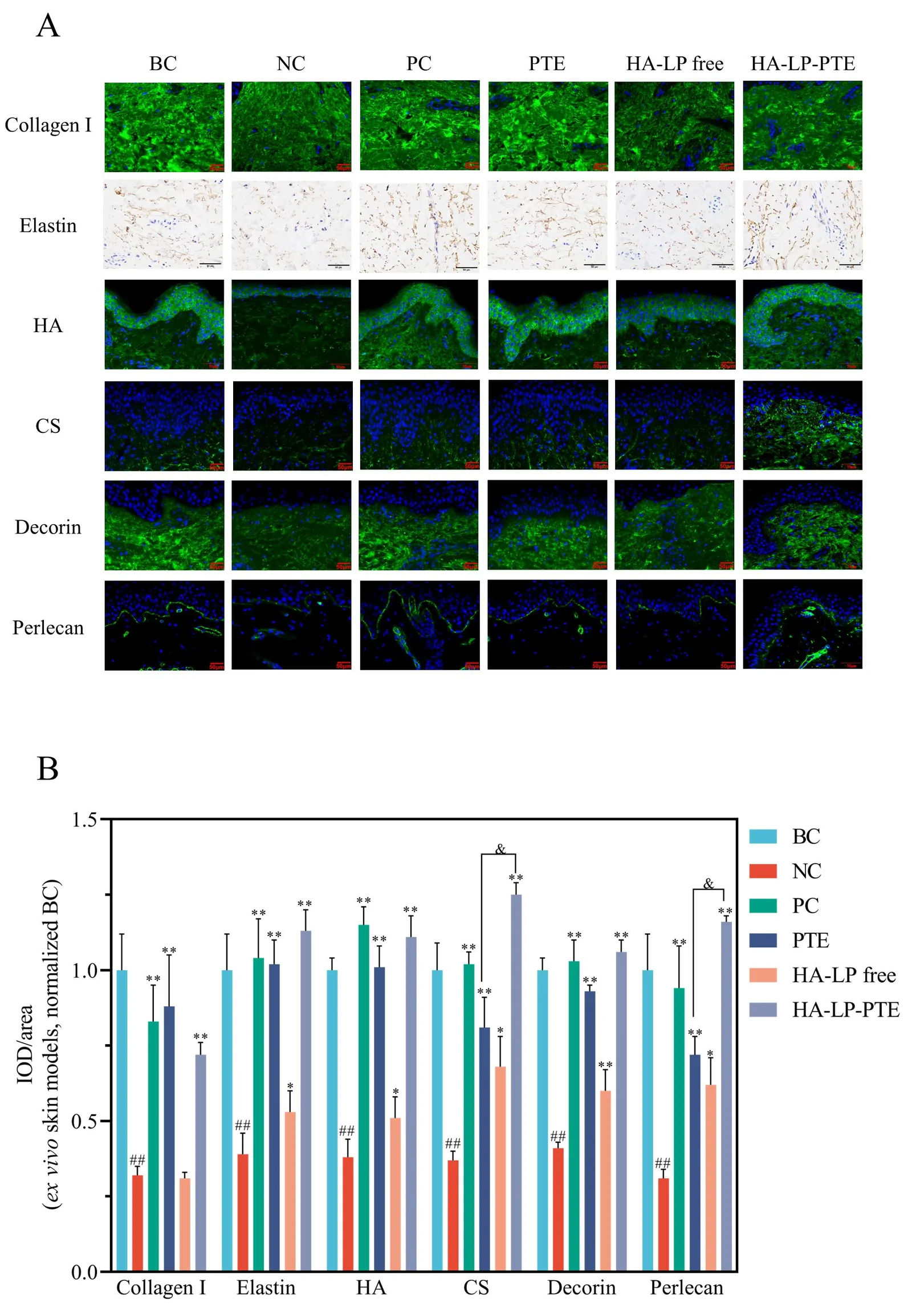
Effects of the formulations on major extracellular matrix (ECM) components in a UV-irradiated ex vivo skin model. (**A**) Representative immunofluorescence and immunohistochemical images of ECM components in skin tissue, including collagen I, elastin, hyaluronic acid (HA), chondroitin sulfate (CS), decorin, and perlecan. The experimental groups were defined as follows: BC, blank control (without UV irradiation); NC, negative control (UV irradiation without active treatment); PC, positive control (UV irradiation with vitamin C and vitamin E treatment); PTE, free pterostilbene; HA-LP free, HA-modified blank liposomes without PTE; and HA-LP-PTE, HA-modified PTE-loaded liposomes. All groups except BC were exposed to UV irradiation. Scale bar: 50 µm. (**B**) Quantitative analysis of ECM components in skin samples. Data are presented as integrated optical density (IOD) divided by the region-of-interest area (IOD/area), which represents the mean staining intensity. One-way ANOVA followed by Dunnett’s post hoc test was used to compare the treatment groups (PTE, HA-LP free, and HA-LP-PTE) with the NC group. Tukey’s HSD post hoc test was used for pairwise comparisons between PTE and HA-LP-PTE and between HA-LP free and HA-LP-PTE. Statistical significance: * *p* < 0.05 and ** *p* < 0.01 versus NC; ## *p* < 0.01 versus BC; & *p* < 0.05 versus PTE.

The effects of the formulations on major ECM components after UV irradiation were evaluated in an ex vivo skin model. The results are shown in Figure 4 and Supplementary Table S3. UV exposure caused large ECM degradation, but the PC prevented much of this loss. Compared with the BC, the NC had lower levels of all six ECM components (all *p* < 0.01). The PC group, treated with vitamins C and E, maintained ECM levels similar to or higher than those in the BC group (0.83–1.15, all *p* < 0.01 vs. NC), confirming that the model worked as expected. HA-LP-PTE also produced strong protective effects, with all ECM component values (0.86–1.25) exceeding those in the NC group (all *p* < 0.01). HA-LP-PTE increased elastin, HA, decorin, CS, and perlecan to levels above those in BC group, suggesting that it may both prevent ECM loss and increase ECM production. Although HA-LP-PTE increased collagen I relative to the NC group, it did not fully restore collagen I to the BC level.

HA-LP free alone increased elastin (0.53 ± 0.07, *p* = 0.035), CS (0.68 ± 0.10, *p* = 0.038), HA (0.51 ± 0.07, *p* = 0.023), perlecan (0.62 ± 0.09, *p* = 0.017), and decorin (0.60 ± 0.07, *p* = 0.038) compared with NC. These findings suggest that the blank HA-containing nanocarrier was not biologically inert. Free PTE gave values of 0.72–1.02 across the six ECM components. HA-LP-PTE showed higher mean values than free PTE for each component. The differences were statistically significant for CS (*p* = 0.022) and perlecan (*p* = 0.007), but not for collagen I, elastin, HA, or decorin (*p* > 0.05).

To test the interaction between the drug and the carrier, the synergy quotient (*Q*) was calculated using a modified Jin’s formula (Supplementary Table S3). A *Q* value≥ 1.15 was defined as synergy, and 0.85 ≤ *Q* < 1.15 was defined as an additive effect. Supplementary Table S3 indicated synergistic effects for CS (*Q* = 1.17) and perlecan (*Q* = 1.18). For perlecan, the net effect of HA-LP-PTE was about 18% greater than the expected additive effect. In contrast, collagen I (*Q* = 0.95), elastin (*Q* = 0.96), HA (*Q* = 0.96), and decorin (*Q* = 0.92) were classified as additive. These calculations suggest that the HA-containing carrier and PTE showed additive contributions to most ECM components and potentially synergistic contributions to CS and perlecan.

### 3.6. Impact of HA-LP-PTE on the Expression of Genes Related to ECM Synthesis

**Figure 5:**
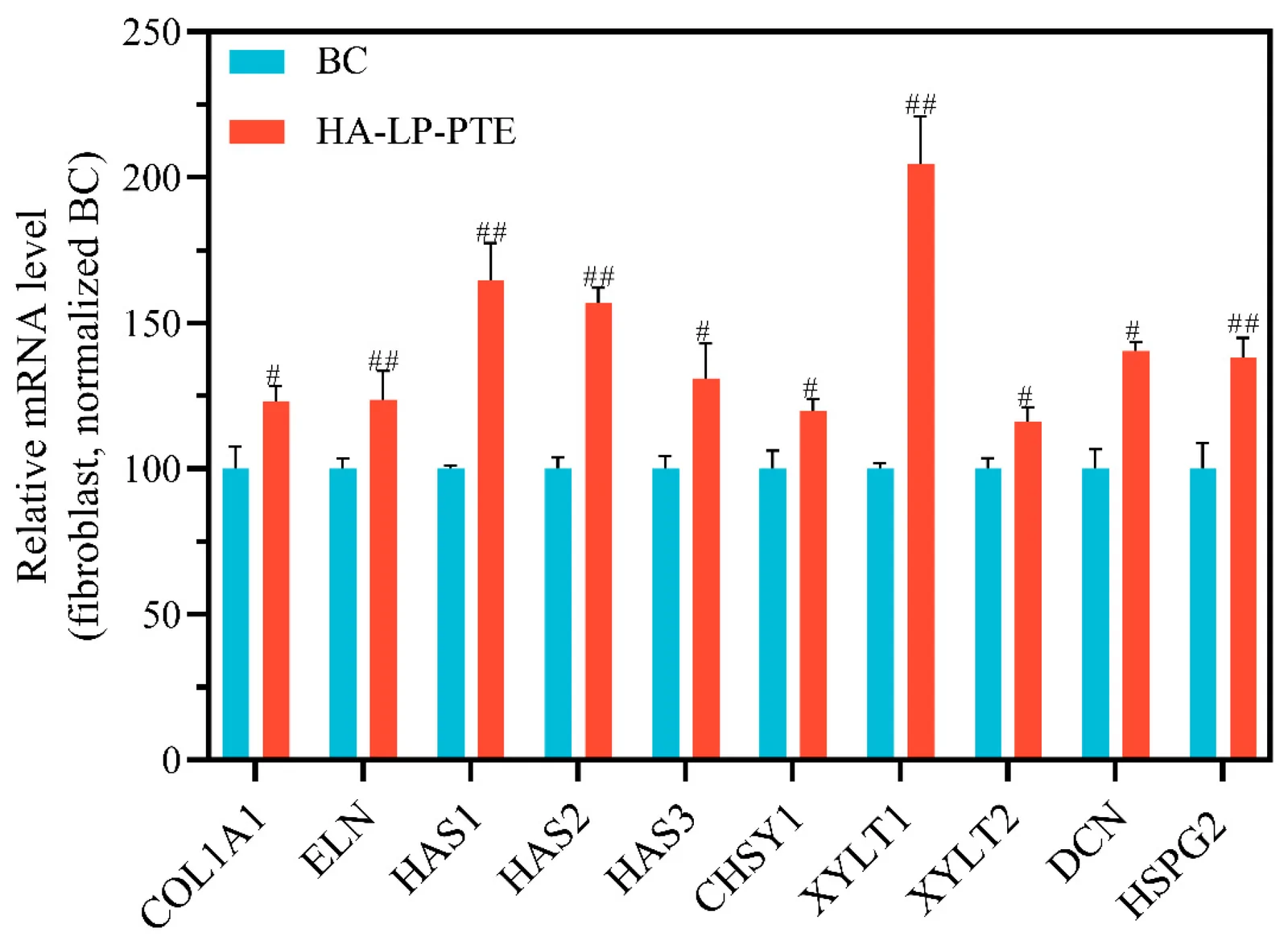
Effects of HA-LP-PTE on the expression of ECM synthesis-related genes in primary human dermal fibroblasts. BC represents the untreated control group. The genes examined included *COL1A1* (collagen type I alpha 1 chain), *ELN* (elastin), *HAS1–3* (hyaluronan synthases 1–3), *CHSY1* (chondroitin sulfate synthase 1), *XYLT1/2* (xylosyltransferases 1 and 2), *DCN* (decorin), and *HSPG2* (heparan sulfate proteoglycan 2). Independent-samples *t*-tests were used to compare gene-expression levels between the BC and HA-LP-PTE-treated groups. Statistical significance: # *p* < 0.05 and ## *p* < 0.01 versus the untreated control.

To explore possible transcriptional correlates of the ECM-related effects of HA-LP-PTE, RT-qPCR was used to measure genes involved in the production of HA, CS, and other PGs. Figure 5 shows that treatment with 0.01% HA-LP-PTE increased the mRNA levels of several ECM-related genes compared with the untreated control. *COL1A1*, *ELN*, *DCN*, and *HSPG2*, which encode collagen I, elastin, decorin, and perlecan, respectively, were all upregulated (*p* < 0.05). Expression of *HAS1–3*, *CHSY1*, *XYLT1*, and *XYLT2*, which participate in GAG and PG synthesis, also increased (all *p* < 0.05).

## 4. Discussion

In this study, HA-modified PTE-loaded nanoliposomes (HA-LP-PTE) were developed to enhance dermal delivery of PTE and its ECM-related effects. LP-PTE and HA-LP free were included as controls to separate the contribution of liposomal encapsulation from that of the HA-containing carrier. Liposomal encapsulation improved PTE delivery, while HA incorporation was associated with greater fibroblast interaction and higher PTE retention in viable skin. HA incorporation also contributed to the observed ECM-related responses. These results provide a basis for further evaluation of HA-LP-PTE as a topical PTE delivery system.

### 4.1. Physicochemical Characteristics of HA-LP-PTE and the Role of HA Modification

Particle size is a critical parameter for nanocarriers because it affects their physicochemical properties, distribution, and ability to penetrate biological barriers [43,44]. Zeta potential reflects the surface charge of nanoparticles and can affect their aggregation behavior through electrostatic interactions [41]. For PTE-loaded nanoliposomes, particle sizes of 16.41–103.3 nm and zeta potentials of −3.56 to −36.2 mV have been reported [18,20,21]. The physicochemical properties of the HA-LP-PTE formulation prepared here fell within these reported ranges. The small particle size may benefit topical delivery because nanoscale vesicles can closely contact the SC and may promote drug deposition in viable skin layers.

Comparing HA-LP free, LP-PTE, and HA-LP-PTE gives more information on the effects of PTE loading and HA incorporation. Within the HA-containing system, PTE loading caused only a small increase in particle size without broadening the particle-size distribution, suggesting that the change was not associated with obvious aggregation. It may reflect PTE intercalation into the hydrophobic region of the lipid bilayer, causing slight membrane expansion or interfacial rearrangement. Studies of resveratrol have shown that stilbene molecules can interact with glycerophospholipid membranes and increase the apparent molecular area of lipid monolayers [45]. Previous studies have also shown that changes in liposome size after phenolic-compound loading depend on the physicochemical properties and concentration of the compound, its location within the liposome, the lipid composition, and the preparation method [43,46]. Therefore, the small increase in HA-LP-PTE particle size may reflect PTE–lipid bilayer interactions rather than vesicle instability.

Comparison with LP-PTE provided further information on the physicochemical changes associated with HA incorporation. HA-LP-PTE showed a modest increase in particle size while maintaining a narrow particle-size distribution. This observation is consistent with changes in the hydrodynamic properties of the liposomal formulation in the presence of HA.

Zeta potential and FTIR data provided complementary information regarding possible HA-associated interfacial changes. HA-LP-PTE exhibited a more negative surface charge than the other formulations, suggesting alterations in the electrokinetic environment of the liposomal formulation. Such changes may arise from differences in interfacial composition and organization, potentially involving interactions among HA, PTE, and the lipid components [47,48]. This explanation agrees with reports that resveratrol in phosphatidylcholine liposomes can alter electrokinetic properties by changing membrane surface charge, interfacial organization, and dipole orientation [48]. FTIR analysis of HA-LP free revealed shifts in HA-related bands and in bands associated with the carbonyl, phosphate, and phosphocholine groups of PtdCho, while the acyl-chain methylene bands showed only minor changes. These observations are consistent with changes in the local chemical environment of the polar lipid groups and suggest possible noncovalent HA–lipid interactions, including hydrogen bonding and electrostatic interactions [49]. However, these measurements do not directly establish the precise structure or spatial distribution of HA at the liposomal surface.

Both PTE-loaded formulations showed high EE and similar mean DL values, which were close to the preset PTE feed concentration of 0.5%. These results show that PTE was successfully encapsulated in the liposomes and that HA modification did not reduce drug encapsulation. The small particle size, low PDI, high EE, and negative zeta potential show that HA-LP-PTE had suitable physicochemical properties for further testing as a dermal PTE carrier.

### 4.2. HA Modification Enhances Fibroblast Association with PTE-Loaded Liposomes

The cellular fluorescence study gave additional evidence that HA incorporation influenced the interaction of nanoliposomes with dermal fibroblasts. At the peak time of 4 h, HA-LP-PTE showed greater cell-associated fluorescence than LP-PTE, suggesting increased cellular association and/or uptake after HA modification. The fluorescence signal of HA-LP-PTE was markedly reduced at 4 °C, consistent with an energy-dependent process. Excess free HA also reduced the signal, supporting a role for HA-associated interactions with fibroblasts. Together with the skin-retention data, these findings suggest that HA incorporation may promote both localization of the nanoliposomes in viable skin layers and interaction with dermal target cells. However, the free-HA competition assay cannot identify the receptor involved or distinguish internalized from surface-bound particles; receptor-specific studies, such as CD44 blocking or knockdown, and direct internalization assays are needed to establish a CD44-mediated uptake mechanism.

### 4.3. HA Modification Enhances Dermal PTE Deposition Beyond Liposomal Encapsulation Alone

Compared with free PTE, liposomal encapsulation increased PTE deposition in the skin. HA modification produced a further increase in the viable epidermis and dermis, with the greatest change in the dermis. Previous PTE-loaded nanoliposomes were reported to increase skin retention by about 1.7–2.34-fold relative to free PTE [18,21]. Although direct cross-study comparisons are limited by differences in experimental design, the enhanced dermal retention achieved with HA-LP-PTE—4.08 times that of free PTE and 1.74 times that of LP-PTE—suggests that HA incorporation may greatly improve dermal deposition.

The formulations also showed different layer-specific retention patterns. LP-PTE showed the highest PTE retention in the SC, whereas HA-LP-PTE accumulated more PTE in the viable epidermis and dermis. Liposomes without HA may therefore be retained preferentially in the lipid-rich SC, while HA modification may favor distribution into deeper viable skin layers. Several factors may contribute to this pattern. HA-associated changes in interfacial properties and local hydration may contribute to the increased deposition of PTE in viable skin. HA-associated interactions with dermal cells may provide another explanation. HA can interact with CD44-expressing cells, including dermal fibroblasts [29,30]. In the present study, HA-LP-PTE showed greater fibroblast-associated fluorescence than LP-PTE, and free HA reduced this signal. Although these findings do not establish a CD44-mediated mechanism, they are consistent with a contribution of HA-associated cellular interactions to dermal localization.

These results show complementary roles for liposomal encapsulation and HA incorporation. Liposomal encapsulation improved the skin deposition of PTE, and HA modification further increased its retention in viable skin, especially in the dermis. Greater dermal deposition may be relevant to the preservation of ECM components under UV-induced stress, as dermal fibroblasts play important roles in ECM synthesis and maintenance.

### 4.4. HA-LP-PTE Restores ECM Homeostasis Through Combined Delivery and Biological Effects

Skin aging is accompanied by progressive changes in ECM composition and organization, including the loss of collagen and elastic fibers and disruption of GAG and PG homeostasis [6]. In the UV-irradiated ex vivo skin model, HA-LP-PTE reduced the loss of all six ECM components examined. The effects were especially apparent for several GAG- and PG-related components. This response indicates that HA-LP-PTE may help preserve selected matrix components under UV-induced damage.

The HA-LP free group helped distinguish the contribution of the carrier from that of PTE. Compared with the UV-damaged negative control, HA-LP free increased elastin, HA, CS, perlecan, and decorin. The blank HA-containing carrier was therefore not biologically inert. This is consistent with the known roles of HA in hydration, tissue repair, and cell signaling [23,24]. HA can also bind cell-surface receptors such as CD44 and may influence pathways involved in matrix synthesis and repair [29,30].

Free PTE partly preserved the ECM after UV irradiation. This effect is compatible with its reported antioxidant, anti-inflammatory, and tissue-repair activities [12,13,14,15], which may reduce oxidative and inflammatory damage to the matrix. HA-LP-PTE produced higher mean values than free PTE for all six ECM components, although significant differences were found only for CS and perlecan. *Q*-value analysis indicated additive effects for collagen I, elastin, HA, and decorin, but synergistic effects for CS and perlecan. This difference suggests that PG-related components may be more responsive to the combined actions of the HA-containing carrier and PTE. PTE may reduce oxidative stress, whereas HA may contribute through hydration and matrix-related signaling. These findings suggest that both PTE and the HA-containing carrier contributed to the ECM response, with a stronger combined effect on CS and perlecan.

Findings from the cellular-association and skin-retention experiments were consistent with this interpretation. At 4 h, fibroblast-associated fluorescence was higher with HA-LP-PTE than with LP-PTE. The signal decreased after free-HA competition and at 4 °C, which is compatible with HA-related interactions and an energy-dependent process. LP-PTE also increased skin deposition of PTE compared with free PTE. HA modification increased retention further in the viable epidermis and dermis. Along with the ECM findings, these results suggest that HA-LP-PTE combines improved local PTE delivery with biological contributions from PTE and the HA-containing carrier. These combined effects may partly explain the ECM responses observed with HA-LP-PTE.

### 4.5. Transcriptional Activation of ECM Synthesis-Related Genes by HA-LP-PTE

The qPCR results further support an ECM-related effect of HA-LP-PTE. Treatment with HA-LP-PTE upregulated genes associated with structural ECM components and GAG and PG synthesis. These transcriptional changes were directionally consistent with the higher ECM-component levels observed in the ex vivo skin model, suggesting that the ECM-related effects of HA-LP-PTE may involve regulation at the transcriptional level.

HA-LP-PTE increased *HAS1–3* and *CHSY1* mRNA levels, consistent with greater transcriptional potential for HA and CS synthesis. *XYLT1* and *XYLT2* were also upregulated. Because these genes act at an early stage of GAG-chain attachment to PG core proteins, their increase may favor PG assembly. A similar pattern was observed in the tissue data, where CS and perlecan levels were higher.

Together, these results show that HA-LP-PTE associated with effects at several levels: increased dermal PTE deposition, HA-associated interactions with dermal fibroblasts, matrix-related support from the HA-containing carrier, attenuation of UV-associated ECM loss, and upregulation of genes involved in ECM synthesis. These combined effects may explain why HA-LP-PTE produced stronger ECM-related responses than free PTE or HA-LP free alone.

### 4.6. Study Strengths and Limitations

A strength of this study is the use of multiple comparison formulations to examine the contribution of individual formulation components. LP-PTE allowed the effect of liposomal encapsulation on PTE skin retention to be assessed, while HA-LP free provided information on the biological activity of the blank HA-containing carrier. This design helped distinguish delivery-related effects from those associated with HA.

However, several limitations should also be considered. Because LP-PTE was not included in the ECM or gene-expression experiments, the separate contributions of HA modification and liposomal encapsulation to the ECM-related effects remain unclear. Including LP-PTE in future UV-induced ECM studies would help distinguish these effects. In addition, the in vitro release kinetics of PTE from HA-LP-PTE and LP-PTE were not evaluated. Free-HA competition reduced the cell-associated fluorescence of HA-LP-PTE, but this assay was not receptor-specific and cannot confirm a direct role for CD44. CD44 blocking or knockdown could clarify whether this receptor contributes to the increased cellular association and dermal localization of HA-LP-PTE. Furthermore, the study relied mainly on ex vivo skin and cultured fibroblasts. These experimental systems do not capture the full in vivo environment. Longer-term safety, irritation potential, persistence of pharmacodynamic effects, and anti-photoaging activity therefore remain to be examined in vivo.

Finally, the preliminary 30-day stability assessment provided information on the physical stability of the formulations under different storage conditions. However, changes in PTE content, encapsulation efficiency, and potential degradation products during storage were not evaluated, nor was the trans-to-cis photoisomerization of PTE directly investigated. Although lipid encapsulation has been reported to improve the chemical stability of certain natural compounds [50,51], whether HA-LP-PTE provides similar protection against PTE isomerization or degradation remains unclear. Further studies incorporating long-term storage assessments, stability-indicating chemical analyses, and controlled light-exposure experiments are needed to comprehensively evaluate the physicochemical and chemical stability of HA-LP-PTE.

## 5. Conclusions

In this study, HA-modified PTE-loaded nanoliposomes were prepared as a topical carrier for dermal PTE delivery. The formulation retained high PTE encapsulation efficiency. Compared with LP-PTE, HA-LP-PTE showed slightly higher fibroblast-associated fluorescence and greater PTE retention in the viable epidermis and dermis. In UV-irradiated skin, HA-LP-PTE increased the measured ECM components, and treatment of dermal fibroblasts upregulated several genes involved in ECM synthesis. Overall, HA modification improved the dermal performance of PTE-loaded liposomes without compromising drug encapsulation, while HA-LP-PTE attenuated UV-associated ECM alterations in the ex vivo skin model. These findings support further development of HA-LP-PTE for localized PTE delivery and continued evaluation of its ECM-protective potential.

## Figures and Tables

**Table 1:** Composition of the liposomal dispersions.

Formulations	PtdCho, %	CCT, %	PTE, %	HA, %
HA-LP free	7	20	/	1
LP-PTE	7	20	0.5	/
HA-LP-PTE	7	20	0.5	1

**Table 2:** Primer sequences used in quantitative real-time PCR analysis and their corresponding sources.

Gene Symbol	Primer Sequence (5′→3′)	Source/Reference
*COL1A1*	CGGCTCCTGCTCCTCTTAG CACACGTCTCGGTCATGGTA	Naranda et al. [35]
*ELN*	GGTGGCTTAGGAGTGTCTGC CACCTACACCTGGAGCCTTG	Dierckx et al. [36]
*HAS1*	CTGCGATACTGGGTAGCCTTCA CCAGGAACTTCTGGTTGTACCAG	OriGene, HP205387 [37]
*HAS2*	GTCATGTACACAGCCTTCAGAGC ACAGATGAGGCTGGGTCAAGCA	OriGene, HP208485 [37]
*HAS3*	AGCACCTTCTCGTGCATCATGC TCCTCCAGGACTCGAAGCATCT	OriGene, HP208486 [37]
*CHSY1*	CGACAGGAACTTTCTCTTCGTGG GGTACAGATGTGTCAGAACCCTC	OriGene, HP211254 [37]
*XYLT1*	TGTCTACGATGAGCCTGACG AAGCGGTCAGCAAGGAAGTA	Shin et al. [38]
*XYLT2*	GGTGGCATTCCTATCCAAGA GTCAGCACGAACCAGTCAGA	Shin et al. [38]
*HSPG2*	CCTGACGGCCACTTCTACC GCATTGTCACATTCACACCCTT	Zhang et al. [39]
*DCN*	GCTCTCCTACATCCGCATTGCT GTCCTTTCAGGCTAGCTGCATC	OriGene, HP205688 [37]
*YWHAZ*	TGATCCCCAATGCTTCACAAG GCCAAGTAACGGTAGTAATCTCC	Zárybnický et al. [40]

The primer sequences for *HAS1*, *HAS2*, *HAS3*, and *CHSY1* are identical to those listed by OriGene Technologies, Inc. (Rockville, MD, USA), under catalog numbers HP205387, HP208485, HP208486, and HP211254, respectively [37].

**Table 3:** Particle size, PDI, zeta potential, EE, and DL of the formulations (Mean ± SD, *n* = 3).

	Particle Size (nm)	PDI	Zeta Potential (mV)	EE (%)	DL (%)
HA-LP free	54.15 ± 1.03	0.157 ± 0.020	−2.49 ± 0.13	/	/
LP-PTE	52.80 ± 0.29	0.089 ± 0.017	−4.73 ± 0.15	97.67 ± 0.75	0.486 ± 0.013
HA-LP-PTE	55.44 ± 0.81	0.120 ± 0.009	−15.12 ± 0.22	99.34 ± 0.51	0.480 ± 0.018

## Data Availability

The data presented in this study are available in this article.

## Supplementary Materials

The following supporting information can be downloaded at https://www.mdpi.com/article/10.3390/pharmaceutics18101313/s1: Figure S1: Representative HPLC chromatograms of total and free PTE in HA-LP-PTE; Table S1: Preliminary physicochemical stability of HA-LP-PTE and LP-PTE under different storage conditions; Table S2: Cell viability of primary human dermal fibroblasts treated with FITC-labeled liposomal formulations; Table S3: Quantitative data from immunofluorescence, immunohistochemistry, and biotin-based staining of key extracellular matrix components depicted in Figure 4, with synergy quotient (Q) analysis.

## Abbreviations

The following abbreviations are used in this manuscript:

ECM	Extracellular Matrix
GAGs	Glycosaminoglycans
PGs	Proteoglycans
HA	Hyaluronic Acid
CS	Chondroitin Sulfate
PTE	Pterostilbene
UV	Ultraviolet
SC	Stratum Corneum
HA-LP free	HA-Modified Blank Liposomes
HA-LP-PTE	Hyaluronic Acid-Modified Pterostilbene-Loaded Liposomes
LP-PTE	Pterostilbene-Loaded Liposomes
PtdCho	Phosphatidylcholine
CCT	Caprylic/Capric Triglyceride
PCR	Polymerase Chain Reaction
TEWL	Transepidermal Water Loss
PDI	Polydispersity Index
TEM	Transmission Electron Microscopy
HPLC	High-Performance Liquid Chromatography
DL	Drug Loading
EE	Encapsulation Efficiency
BC	Blank Control
NC	Negative Control
PC	Positive Control
COL1A1	Collagen Type I Alpha 1 Chain
ELN	Elastin
HAS1-3	Hyaluronan Synthases 1–3
CHSY1	Chondroitin Sulfate Synthase 1
XYLT1/2	Xylosyltransferases 1/2
DCN	Decorin
FTIR	Fourier Transform Infrared Spectroscopy
