# Supplementary material for: Multifunctional Hyaluronic Acid-Modified Pterostilbene Nanoliposomes for Enhanced Dermal Delivery and Extracellular Matrix Protection
Source: Pharmaceutics. 2026 Oct 16;18(10):1313. doi: 10.3390/pharmaceutics18101313 (PMC13708579; doi:10.3390/pharmaceutics18101313)
Supplement: Supplementary file 1 [file pharmaceutics-18-01313-s001.zip › Supplement Figure S1.pdf]

Figure S1

A

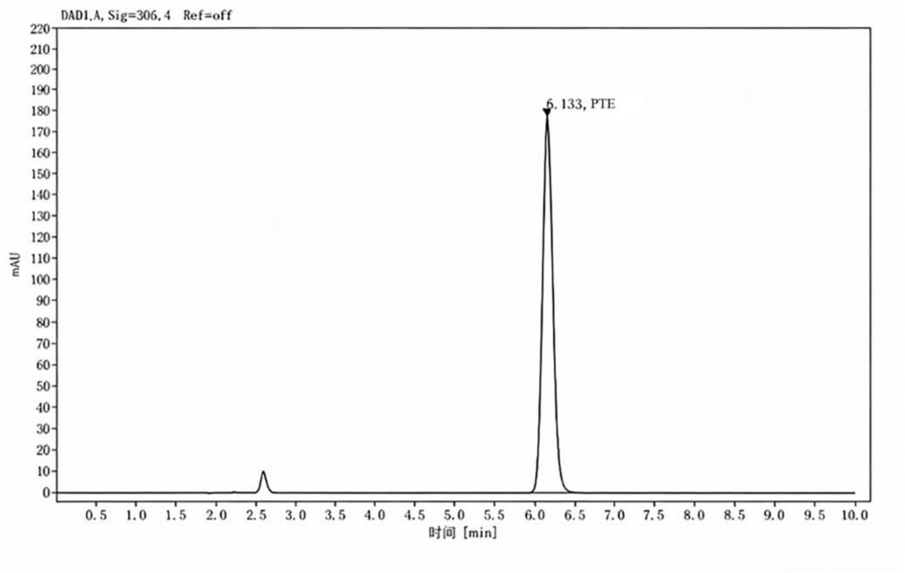

B

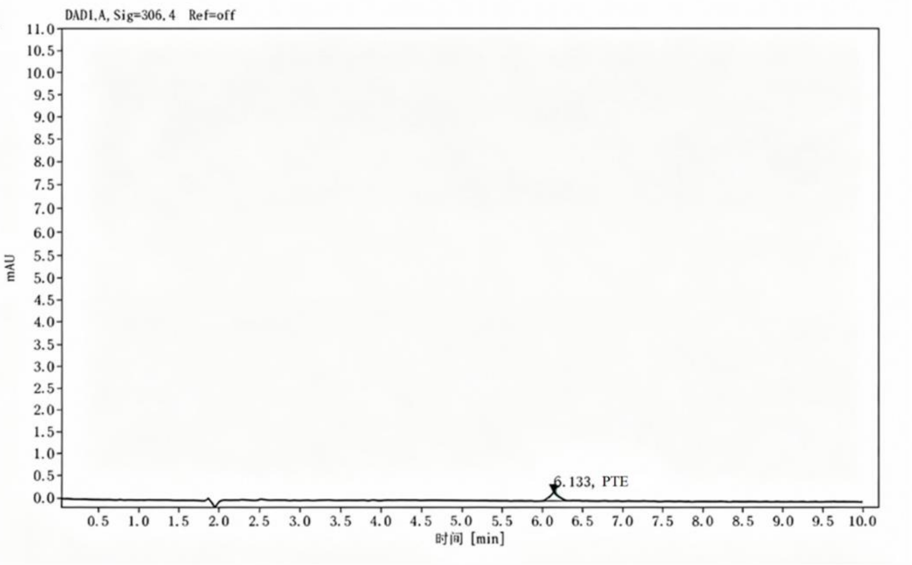

Figure S1. Representative original HPLC chromatograms used for the determination of encapsulation efficiency (EE) and drug loading (DL) of HA-LP-PTE. (A) Chromatogram of total pterostilbene (PTE; W_total) in the HA-LP-PTE formulation. (B) Chromatogram of free PTE (W_free) in the ultrafiltrate obtained by ultrafiltration–centrifugation. PTE was detected at 306 nm, with a retention time of approximately 6.13 min. The integrated peak areas were used to quantify W_total and W_free and calculate EE and DL according to Equations (1) and (2), respectively.
